# Information Extraction from Lumbar Spine MRI Radiology Reports Using GPT4: Accuracy and Benchmarking Against Research-Grade Comprehensive Scoring

**DOI:** 10.3390/diagnostics15070930

**Published:** 2025-04-04

**Authors:** Katharina Ziegeler, Virginie Kreutzinger, Michelle W. Tong, Cynthia T. Chin, Emma Bahroos, Po-Hung Wu, Noah Bonnheim, Aaron J. Fields, Jeffrey C. Lotz, Thomas M. Link, Sharmila Majumdar

**Affiliations:** 1 Department of Radiology and Biomedical Imaging, University of California San Francisco, San Francisco, CA 94143, USA; 2 Department of Bioengineering, University of California Berkeley, Berkeley, CA 94720, USA; 3 Department of Bioengineering, University of California San Francisco, San Francisco, CA 94143, USA; 4 The UCSF REACH Center, The Core Center for Patient-Centric Mechanistic Phenotyping in Chronic Low Back Pain, San Francisco, CA 94143, USA; 5 Department of Orthopaedic Surgery, University of California San Francisco, San Francisco, CA 94143, USA

**Keywords:** large language models, spinal imaging, magnetic resonance imaging

## Abstract

**Background/Objectives**: This study aimed to create a pipeline for standardized data extraction from lumbar-spine MRI radiology reports using a large language model (LLM) and assess the agreement of the extracted data with research-grade semi-quantitative scoring. **Methods**: We included a subset of data from a multi-site NIH-funded cohort study of chronic low back pain (cLBP) participants. After initial prompt development, a secure application programming interface (API) deployment of OpenAIs GPT-4 was used to extract different classes of pathology from the clinical radiology report. Unsupervised UMAP and agglomerative clustering of the pathology terms’ embeddings provided insight into model comprehension for optimized prompt design. Model extraction was benchmarked against human extraction (gold standard) with F1 scores and false-positive and false-negative rates (FPR/FNR). Then, an expert MSK radiologist provided comprehensive research-grade scores of the images, and agreement with report-extracted data was calculated using Cohen’s kappa. **Results**: Data from 230 patients with cLBP were included (mean age 53.2 years, 54% women). The overall model performance for extracting data from clinical reports was excellent, with a mean F1 score of 0.96 across pathologies. The mean FPR was marginally higher than the FNR (5.1% vs. 3.0%). Agreement with comprehensive scoring was moderate (kappa 0.424), and the underreporting of lateral recess stenosis (FNR 63.6%) and overreporting of disc pathology (FPR 42.7%) were noted. **Conclusions**: LLMs can accurately extract highly detailed information on lumbar spine imaging pathologies from radiology reports. Moderate agreement between the LLM and comprehensive scores underscores the need for less subjective, machine-based data extraction from imaging.

## 1. Introduction

Low back pain (LBP) is one of the major causes of disability worldwide [1], with a substantial impact on both quality of life for affected patients and healthcare expenditures, which outpace the rate of inflation [2]. One of the most deployed tools in the diagnostic work-up of LBP is lumbar spine magnetic resonance imaging (MRI). While this diagnostic tool can demonstrate pathologies that may require surgical intervention, many scans do not uncover any addressable pathology or plausible culprit lesion linked to clinical symptoms [3]. One strategy to address this knowledge gap between imaging findings and clinical outcomes is to investigate large databases, e.g., institutional electronic healthcare records. However, the considerable resources needed to extract standardized and clinically relevant data from patient electronic health records at a multi-institution scale has historically limited “big data” studies. Radiology reports are a potential additional source of pre-existing image-derived data, yet these reports are mainly in the form of free-text, which is not amenable to established biostatistical or epidemiologic analytic approaches.

With the advent of modern large language models (LLMs), standardized information extraction from clinical radiology reports may be feasible, and has been successfully piloted in several fields [4,5], notably for malignant diseases of the liver [6], breasts [7], and lungs [8]. Radiology reports, often narrative in format, contain valuable details about pathology, patient history, and diagnostic impressions, but their unstructured nature poses challenges for direct data extraction and analysis. Modern LLMs use attention mechanisms to efficiently capture complex dependences across short contexts (i.e., adjectives modifying nouns) and long contexts (i.e., pronouns referring to nouns). The numerical representation of input text, referred to as text embeddings, offers valuable insight into the model’s processing. In the model encoder, LLMs perform tokenization by breaking text down into words or characters, and then transform these tokens into embeddings, which are continuous vectors that capture semantic meaning. These embeddings provide a structured, comparable data format and another avenue to evaluate the model’s semantic accuracy in understanding medical terminology. These embeddings are used in the model decoder for next word prediction, allowing us to assess the reliability of the extractions against expert-provided labels.

While LLM information extraction pipelines show promising performance when benchmarked against manual data extraction, comparisons with research-grade scores, i.e., comprehensive qualitative or semi-quantitative scoring of images, are lacking. A mismatch between clinical reports and research-grade comprehensive scoring is expected, as the tasks differ somewhat in their general aim. However, when considering the potential utility of clinical radiology reports for research purposes, benchmarking report-derived data against expert, research-grade scoring is required to provide guidance on the strengths and limitations of this data source. Thus, this study aimed to first create a pipeline for the accurate extraction of standardized pathological findings from lumbar spine MRI radiology reports using an LLM, and then assess the agreement of the extracted data with comprehensive and semi-quantitative expert scoring.

## 2. Materials and Methods

### 2.1. Study Population and Imaging

A subset of 230 participants who participated in the HIPAA-compliant, IRB-approved Longitudinal Clinical Cohort for Comprehensive Deep Phenotyping of Chronic Low-Back Pain (cLBP) in Adults Study (comeBACK) [9], a part of the NIH HEAL Initiative’s Back Pain Consortium (BACPAC) Research Program [10], were included in this analysis. To be eligible for inclusion in this post hoc analysis, participants must have had an available full clinical radiology report and comprehensive image scoring. Of the 450 participants enrolled in comeBACK, 239 had comprehensive MRI scores from their baseline scan at the time of this analysis and *n* = 9 of them were excluded due to unavailable clinical radiology reports. The mean patient age was 53.2 ± 15.3 years and 54.3% (125/230) of the patients were women. All participants underwent a 3.0 T non-contrast lumbar spine MR scan at baseline [11]. The MR acquisitions, which were read by clinical radiologists and scored by experts, included standardized sagittal T2-weighted fat saturated, sagittal and coronal T1-weighted, axial T1-weighted, and axial T2-weighted images covering the region between the vertebrae T12 and S1 [12]. All participants gave written informed consent before their inclusion in the study.

### 2.2. Prompt Engineering and LLM Pipelines

The LLM used in this analysis was a protected health information (PHI)-compliant deployment of GPT-4 (8-k model, OpenAI, San Francisco, CA, USA; Microsoft Azure, Microsoft, Redmond, WA, USA); under the name VERSA, this deployment can be used both with a chat interface and an application programming interface (API) [6]. For our pipeline, VERSA-API was used within a Python script, which is publicly available (https://github.com/michelle-tong18/VERSA-spine, version 1.0, accessed on 16 March 2025).

**Figure 1:**
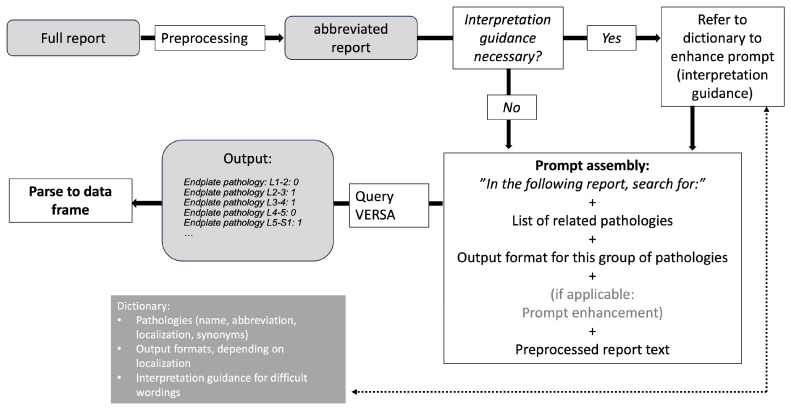
Simplified pipeline schematic. Preprocessing: deletion of unneeded text (e.g., date of the exam, reporting physician, etc.). Interpretation guidance: a simple search logic determines whether challenging wordings, identified in initial prompt development, are present.

In an initial exploration, prompting strategies were tested on ten representative reports not used in this analysis. For preprocessing, unnecessary text passages (e.g., scan date) and the word “significant” were removed, as it led to erroneous detection in the context of “no significant stenosis”, which was consistently understood to imply stenosis. Preprocessed text was then fed into the LLM, and the prompts were refined until information extraction on the ten sample reports showed >90% agreement with human interpretation. From this process, few-shot prompting (i.e., providing specific examples of desired output within the prompt text) emerged as the most effective strategy. Briefly, a prompt is assembled from predefined building blocks, adding information on pathology as well as the format of the output and instructions on how to handle ambiguous wording (if present) (see Figure 1). The assembled prompt is then fed into the large language model, and the output is parsed into a data frame in a fully automated process; an example extraction is then given as Supplementary Figure S1. Additional hyperparameters were set to improve the generation of relevant responses, which should be purely numeric: the output token limit was set to 50 to limit unwanted verbosity and the temperature was set to 0 to decrease the chance of hallucinations [13]. The used tokens, separated into input and output tokens, were extracted as secondary output and used to calculate the cost of processing per report.

### 2.3. Comprehensive Image Scoring and Manual Report Data Extraction

Comprehensive semi-quantitative image scoring included the assessment of a range of pathologies with relevance to cLBP [12]; for the purpose of this investigation, only assessments of pathologies routinely included in the semi-structured clinical reports. Table 1 shows the included scoring items. Briefly, the items included disc abnormalities (bulging, protrusion, and extrusion), endplate changes (endplate erosions, Schmorl’s nodes, and Modic changes), central spinal canal stenosis, lateral recess stenosis, foraminal stenosis, and facet joint arthropathy at each spinal level (L1 through S1). Laterality (left versus right) was noted for lateral recess stenosis and foraminal stenosis. Lastly, the presence of sacroiliac joint changes, alterations in spinal curvature, or the presence of a vertebral fracture were noted. All images were scored by an expert musculoskeletal radiologist (TML). Data of the same structure, as detailed in Table 1 were manually extracted from anonymized clinical radiology reports. Pathologies not mentioned were graded as absent. All reports were manually processed by two trained radiologists (KZ and VK), independently and without access to underlying imaging or other patient-related information; cases of disagreement were adjudicated by a third radiologist (CTC).

### 2.4. Semantic Information Representation

Text embeddings were generated for each pathology term to capture domain-specific content for the assessment of semantic closeness. The “text-embedding-ada-002” model (OpenAI, Microsoft Azure), which was optimized for versatile tasks, tokenized the input text into smaller semantic units (tokens) using “cl100k_base”, and extracted their numerical representation into a vector of a length of 1536 from the model’s embedding layer. Unsupervised uniform manifold approximation projections (UMAPs) [14,15] and cosine similarity with hierarchical clustering [16] of embeddings for pathology synonyms were created to visualize term similarity, motivating the model’s language comprehension. Through a grid search, the optimal UMAP parameters for the number of neighbors, minimum distance, negative sample rate, and local connectivity were selected to minimize the cluster silhouette score. For cosine similarity visualization, agglomerative clustering iteratively merged the closest clusters based on the distance between rows and columns to capture term closeness in the heatmap.

### 2.5. Statistical Analysis

Human-extracted report information served as the reference standard, against which the model performance was measured. The F1 score, false-positive rate (FPR), and false-negative rate (FNR) were computed for each imaging finding separately. Ordinal measures were collapsed to binary measures (0 = absent/all other = present) for this analysis. In the second step, the agreement of report-extracted information from VERSA-spine with comprehensive image scoring was calculated. For this, Cohen’s kappa values were computed for each pathology and location, and the mean kappa values with ranges were reported per pathology. Ordinal measures were analyzed with weighted kappa with linear weights [17]. As an advantage over percent agreement, Cohen’s kappa accounts for agreement by chance. Kappa values are commonly interpreted according to Landis and Koch [18] as follows: 0.00–0.20 slight agreement; 0.21–0.40 fair agreement; 0.41–0.60 moderate agreement; 0.61–0.80 substantial agreement; and 0.81–1.00 almost perfect agreement. All analyses were performed using the Python version 3.11.8. Statistical significance was set to *p* < 0.05.

## 3. Results

### 3.1. LLM Performance

Using the adjudicated human report interpretation as the standard of reference, the VERSA-spine pipeline showed excellent performance. The results of individual diagnostic performance per pathology class are given in Table 2. The mean F1 score across all pathology classes was 0.960, with the best performance seen in pathologies assessed only at the level of the whole patient—sacroiliac joint pathology and scoliosis, both with F1 scores of 0.987. Notably, the mean FPR was slightly higher than the FNR at 5.1% vs. 3.0%. Taking a closer look at the individual pathologies, disc pathologies had the highest false-positive rates at 16.8%, while endplate pathologies had the highest false-negative rates at 13.6%.

The average token cost of VERSA-spine, which extracts a total of 42 individual data points from each report, was 1967.4, which translates into an approximate cost of $0.06 per report using the 8-k model at the time of manuscript preparation (September 2024).

### 3.2. LLM Embedding Representation of Semantic Information

**Figure 2:**
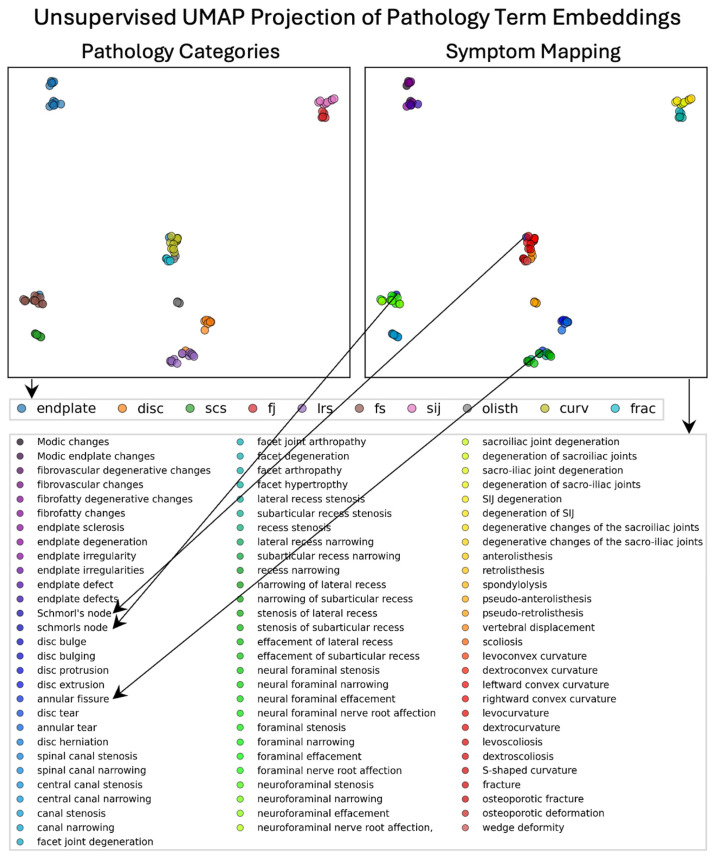
Unsupervised UMAPs of embeddings of pathology terms. SCS = spinal canal stenosis. LRS = lateral recess stenosis. FS = foraminal stenosis. SIJ = sacroiliac joint. Curv = pathology of the curvature of the spine. Olisth = olisthesis. Frac = fracture. The contextual specificity of pathology term embeddings are observed: Joint pathology (“facet”—red and “sacroiliac”—pink) and stenosis pathology (“spinal canal”—green and “lateral recess”—brown) form closely related clusters that are distinct and separate from other clusters. Three terms indicated by arrows (“schmorl’s node”, “schmorls node”, and “annual fissure”) were misclassified suggesting, that they were challenging terms for the model.

**Figure 3:**
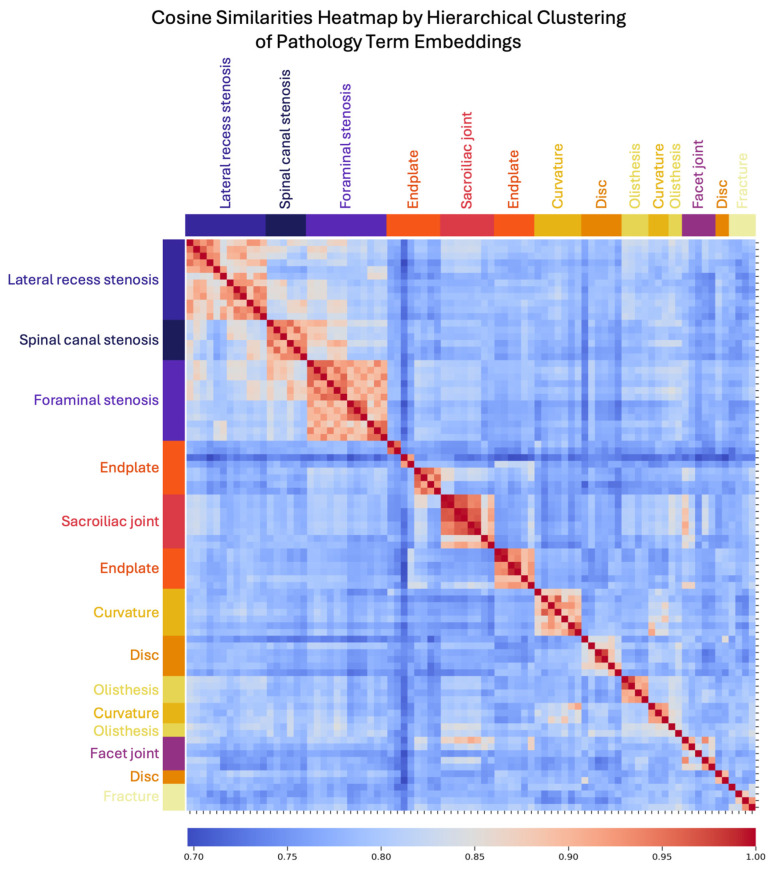
Heatmap of cosine similarity between embeddings of pathology terms. Cosine similarities (red–blue) between each pair of pathology term embeddings are shown in the heatmap, with values of 1 indicating perfect similarity. Unsupervised agglomerative clustering of cosine similarities allowed for a data-driven approach to understand model comprehension of term similarity. The color bar along the *y*-axis shows expert assigned pathology categories in comparison with data-derived categories. Distinct color bands (yellow–purple), such as for each type of stenosis, suggest that the model can reconcile semantic variability when addressing the challenge of diverse medical terminology, thereby lending insight for prompt development.

The embeddings generated effectively captured semantic relationships between pathological terms, as evident from the formation of distinct clusters after UMAP dimensionality reduction (number of neighbors = 10, min. distance = 0.01, negative sample rate = 6, local connectivity = 3) and agglomerative clustering of the cosine similarity between terms. Pathological findings such as “spinal canal stenosis” formed a cluster distinct from related terms like “foraminal stenosis”, demonstrating the model’s ability to differentiate between clinically distinct yet related terms. Unsupervised UMAPs formed well-defined clusters, with a silhouette score of 0.64 shown in Figure 2. Clusters for joint pathologies were distinct yet appropriately related, reflecting the model’s nuanced understanding of clinical terminology. These results validate the embeddings’ contextual integrity despite GPT-4.0’s development for general purpose usage, underscoring their utility for accurate information retrieval from unstructured radiology reports. A further evaluation of the embedding quality in Figure 3 shows a heatmap and dendrogram of the cosine similarity between pathology terms (detailed axes specifying pathology terminology and dendrogram clustering are included in Supplementary Figure S2). Distinct clusters emerged for stenosis types, supporting the model’s differentiation capabilities.

### 3.3. Agreement of Report-Derived Data with Comprehensive Image Scoring

The agreement of VERSA-spine with comprehensive expert scoring, as well as of FPR and FNR with comprehensive scores as a standard of reference, are given in Table 3.

**Figure 4:**
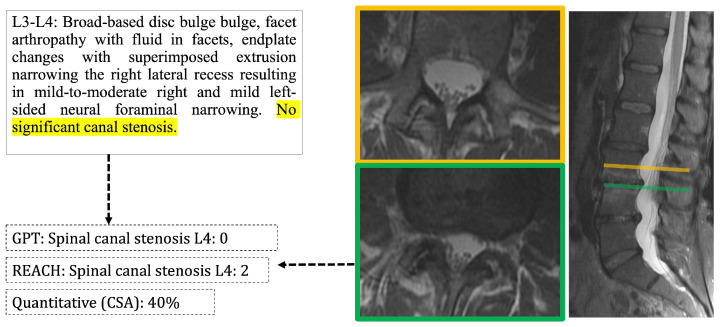
Example of disagreement between comprehensive scores and reports. The clinical report (**top left**) indicates no significant stenosis (section highlighted in yellow), thus the LLM (= GPT) finds no stenosis for this level. The comprehensive score (REACH) detects moderate stenosis. Manual quantification shows 40% narrowing when compared with mid-vertebral canal width at L3 (orange line and orange-framed axial section).

The mean kappa scores indicate a moderate agreement of 0.424, but values differed substantially between pathologies. The lowest agreement with comprehensive scoring was found for sacroiliac joint pathology (kappa 0.234) and the highest for foraminal stenosis (0.653). Generally, underreporting was a greater source of disagreement than overreporting, shown by a mean FPR of 18.6% vs. a mean FNR of 36.6%. Furthermore, the source of disagreement differed between pathologies; while disc pathology tended to be overreported in the clinical reports, with an FPR of 42.7%, lateral recess stenosis was underreported, with an FNR of 63.6%. A specific example of disagreement is given in Figure 4.

## 4. Discussion

Our study aimed to develop an information extraction pipeline for clinical lumbar spine radiology reports and to compare the extracted data with comprehensive expert semi-quantitative scoring, which is the current research standard for analyzing imaging pathologies. The proposed pipeline showed excellent performance in information extraction, but limitations in agreement with comprehensive scoring were noted.

The extraction performance of our pipeline was excellent, with a mean F1 score across different pathologies of 0.960, which can compete with the values reported in the literature for related tasks, ranging from 0.688 [19] to 0.980 [20]. The extracted data have a high level of anatomical detail (e.g., not just general presence/absence of foraminal stenosis, but specific grades for each lumbar foramen), and performance was similar for different classes of pathologies. This higher level of anatomical detail differentiates our work from that of previous studies that employed older, custom-built natural language processing systems [21]. Agreement with comprehensive image scoring was moderate, but not substantially lower than agreement between different radiologists performing similar tasks [22]. Apart from data mining for retrospective analysis, these technologies may also be deployed to provide ground truth for automated image analysis studies in a more resource effective manner.

One of the main challenges in extracting information from routine clinical data for scientific use is the fundamentally different context in which radiologists annotate the images. A comprehensive and structured scoring system aims to capture both normal and pathological findings, regardless of clinical symptoms, to which the reader is often blind. While this measure is taken to ensure objectivity, it may also introduce challenges in the correct interpretation of equivocal findings [23]. A clinical radiology report, on the other hand, aims to identify a pathology that may explain present symptoms. Despite a trend towards structured reporting in radiology [24], the fact remains that the satisfaction of search errors [25], neglect of minor pathology, heterogeneous levels of reader experience, time pressure, and exhaustion [26] inherently impact the quality and quantity of data in clinical radiology reports. Reporting practices may vary considerably between institutions, yet taking note of this limitation in the setup of larger database query studies with LLMs can improve the interpretability of the extracted data, e.g., by performing benchmarking exercises on smaller samples.

Historically, logic-based attempts at extracting information from free text required advanced programming experience and exhibited variable levels of performance [27]. Current advancements in LLMs may help to perform tasks with prompts that only require language understanding and logic, which makes this technology accessible to a much larger group of researchers in healthcare [28]. Furthermore, such prompts can be shared easily between researchers. Still, prompt engineering often requires an iterative approach that incorporates considerations of medical domain-specific nuances such as risk-adverse documentation to elicit accurate responses. As an example from our investigation, ambiguous wordings like “multilevel disc bulging”, were handled as “affecting all levels”; this strategy may overestimate the actual changes, and in part explain the relatively high number of false positives for this specific pathology.

This study highlights the utility of LLMs in generating high-quality embeddings for extracting structured information from unstructured radiology reports. Pathology terminology was grouped into distinct clusters observed in UMAPs, demonstrating the model’s ability to capture clinically meaningful relationships between terms. These findings were further validated through clear association in the cosine similarities between similar terms, which quantified the model’s comprehension of medical terminology for this domain-specific extraction task. By effectively distinguishing between related yet distinct terms, such as types of stenosis, these embeddings align closely with clinical reasoning and can reveal discrepancies between model and expert comprehension, a critical consideration for downstream applications of medical text analysis. This supports the optimized prompt design, which only mentioned the primary pathology and excluded synonyms stemming from variations in language, as an effective strategy to reduce token usage and its associated costs without compromising performance.

Our findings underscore the potential of LLM-derived embeddings to enhance prompt design and improve information retrieval for medical texts by providing insight into model comprehension. Transforming unstructured text into structured, comparable representations enables the quantification of the text’s semantic proximity in high-dimensional space and the validation of the embedding’s quality to reflect intuitive, clinically accurate relationships. Future efforts in prompt optimization can leverage embedding space analyses to identify challenging terminology or reports, refine prompts, and characterize possible model limitations.

Our study had certain limitations that affect the generalizability of our findings. Firstly, our analysis included reports from a single institution—this allowed for a highly specific and therefore accurate prompt development, but achieving similar accuracy may be more challenging when analyzing reports from different institutions. Furthermore, the selection of pathologies to be extracted from clinical reports is somewhat subjective, and other authors may choose different pathologies to include or exclude. Another limitation regarding the widespread deployment of LLMs to medical record data revolves around concerns about data confidentiality. In our study, we used a PHI-compliant deployment of OpenAI’s GPT-4. Recently, many non-proprietary LLMs with different model weights and underlying training data have become available, with somewhat differing accuracies in radiology applications [29]. Some of these models can be set up to run locally, further limiting the possibility of the leakage of confidential information [30]. Another limitation of commercial LLMs may be the cost associated with their use. In our investigation, we used a highly detailed extraction strategy with associated costs of $0.06 per processed report. While these prices are small, and certainly much smaller than the cost of having trained specialists performing similar tasks, they may still limit the deployment of these technologies outside larger research centers.

## 5. Conclusions

In conclusion, LLMs can be leveraged to reliably extract highly detailed information on lumbar spine imaging pathologies from radiology reports, enabling research studies that encompass large-scale clinical databases, opening up opportunities for advancements in the understanding of the complex interplay between different imaging pathologies in LBP. The moderate agreement with comprehensive scoring in the reported ranges of agreement between different radiologists in such frameworks suggests a role for less subjective, more quantitative, and machine-based methods of extracting pathological features from imaging in the context of LBP research.

## Figures and Tables

**Table 1:** Extracted data points. Location = anatomical site at which pathology is assessed; level = assessed at each intervertebral level from L1–L2 to L5–S1; level and side = assessed at each intervertebral level for left and right side separately; and overall = assessed per patient. Scale = level of detail in which the pathology is assessed.

Pathology Class	Location	Scale	Included Pathologies
Endplate pathology	level	absent/present	Endplate erosions, Schmorl’s nodes, Modic changes
Disc pathology	level	absent/present	Bulging, protrusion, extrusion
Facet joint arthropathy (FJ-OA)	level	absent/present	Hypertrophic changes, synovial cysts, increased facet joint fluid
Lateral recess stenosis (LRS)	level and side	absent/present	Nerve root contact, compression
Spinal canal stenosis (SCS)	level	absent, mild, moderate, severe	Varying grades of stenosis, regardless of cause
Foraminal stenosis	level and side	absent, mild, moderate, severe	Varying grades of stenosis, regardless of cause
Sacroiliac joint (SIJ) pathology	overall	absent/present	Any observed joint changes, including sclerosis, bone marrow signal changes (edema/fat)
Scoliosis	overall	absent/present	Changes in curvature of lumbar spine

**Table 2:** VERSA-spine performance. CI = confidence interval. FPR = false-positive rate. FNR = false-negative rate. LL = lower limit. UL = upper limit. Acc = accuracy. FJ-OA = facet joint osteoarthritis. LRS = lateral recess stenosis. SCS = spinal canal stenosis. SIJ path = sacroiliac joint pathology.

Pathology	F1	F1: 95% CI		FPR	FPR: 95% CI		FNR	FNR: 95% CI		Acc.	Acc: 95% CI	
		LL	UL		LL	UL		LL	UL		LL	UL
Endplate	0.949	0.939	0.958	0.039	0.033	0.047	0.136	0.089	0.172	0.948	0.933	0.959
Disc	0.938	0.930	0.948	0.168	0.111	0.243	0.015	0.009	0.023	0.951	0.937	0.962
FJ-OA	0.957	0.938	0.974	0.006	0.006	0.006	0.035	0.035	0.035	0.981	0.971	0.987
LRS	0.970	0.957	0.981	0.004	0.004	0.004	0.000	0.000	0.000	0.929	0.917	0.939
SCS	0.921	0.898	0.950	0.109	0.064	0.155	0.018	0.002	0.042	0.923	0.906	0.937
FS	0.970	0.966	0.974	0.053	0.038	0.068	0.008	0.003	0.013	0.974	0.967	0.980
SIJ path.	0.987	0.987	0.987	0.018	0.018	0.018	0.000	0.000	0.000	0.991	0.969	0.998
Scoliosis	0.987	0.987	0.987	0.007	0.007	0.007	0.025	0.025	0.025	0.991	0.969	0.998
Mean	0.960			0.051			0.030			0.961		

**Table 3:** Agreement with comprehensive scoring. CI = confidence interval. FPR = false-positive rate. FNR = false-negative rate. * = ordinal variables that were binarized to present/absent to calculate FPR and FNR, and for which the reported kappa is weighted (linear).

Pathology	Kappa	F1: 95% CI		FPR	FPR: 95% CI		FNR	FNR: 95% CI	
		Lower	Upper		Lower	Upper		Lower	Upper
Endplate	0.454	0.406	0.504	0.043	0.026	0.065	0.557	0.485	0.620
Disc	0.495	0.410	0.587	0.427	0.289	0.557	0.082	0.064	0.109
Facet arthropathy	0.341	0.248	0.439	0.135	0.135	0.135	0.441	0.441	0.441
Lateral recess stenosis	0.286	0.246	0.329	0.014	0.014	0.014	0.636	0.636	0.636
Spinal canal stenosis *	0.652	0.483	0.774	0.129	0.056	0.213	0.267	0.157	0.405
Foraminal stenosis *	0.653	0.616	0.693	0.241	0.155	0.328	0.197	0.144	0.258
SIJ pathology	0.234	0.234	0.234	0.215	0.215	0.215	0.556	0.556	0.556
Scoliosis	0.278	0.278	0.278	0.284	0.284	0.284	0.192	0.192	0.192
Mean	0.424			0.186			0.366		

## Data Availability

The data underlying this article cannot be shared publicly due to the privacy concerns of individuals participating in the study. The data will be shared upon reasonable request to the corresponding author. All underlying codes, including LLM prompts, have been made publicly available in full (https://github.com/michelle-tong18/VERSA-spine, accessed on 16 March 2025).

## Supplementary Materials

The following supporting information can be downloaded at: https://www.mdpi.com/article/10.3390/diagnostics15070930/s1. Figure S1. Prompt assembly and pipeline schematic. Preprocessing: Crossed out text is deleted (red). A simple search logic determines whether challenging wordings, identified in initial prompt development are present (purple) – in this example, the interpretation guidance that was used in the later prompt is framed in red. Main instruction: three different prompts are generated for each report, one for level-wise, one for level- and side-wise and one for patient-wise (green) pathologies (orange) with formatting instruction (dark blue) and few-shot examples (light blue). These are fed sequentially to VERSA, and their respective outputs are then parsed into a data frame. Figure S2. Heatmap of Cosine Similarity between Embeddings of Pathology Terms. Scs = spinal canal stenosis. Lrs = lateral recess stenosis. Fs = foraminal stenosis. Sij = sacroiliac joint. Curv = pathology of the curvature of the spine. Olisth = olisthesis. Frac = fracture. Cosine similarities (red-blue) between each pair of each pathology term embeddings are shown in the heatmap with values of 1 indicating perfect similarity. Unsupervised agglomerative clustering of cosine similarities allowed for a data driven approach to understand model comprehension of term similarity. The color bar along the y-axis shows expert assigned pathology categories in comparison with data-derived categories. Distinct color bands (yellow-purple), such as for each type of stenosis, suggest the model can reconcile semantic variability when addressing the challenge of diverse medical terminology, thereby lending insight for prompt development.

## Abbreviations

The following abbreviations are used in this manuscript:

API	Application programming interface
cLBP	Chronic low back pain
FNR	False-negative rate
FPR	False-positive rate
LLM	Large language model
PHI	Protected health information
UMAP	Unsupervised uniform manifold approximation projections
