# Supplementary material for: Information Extraction from Lumbar Spine MRI Radiology Reports Using GPT4: Accuracy and Benchmarking Against Research-Grade Comprehensive Scoring
Source: Diagnostics (Basel). 2025 Apr 4;15(7):930. doi: 10.3390/diagnostics15070930 (PMC11989208; doi:10.3390/diagnostics15070930)
Supplement: Supplementary file 1 [file diagnostics-15-00930-s001.zip › diagnostics-3560773-supplementary/Supplementary_Figure1.pdf]

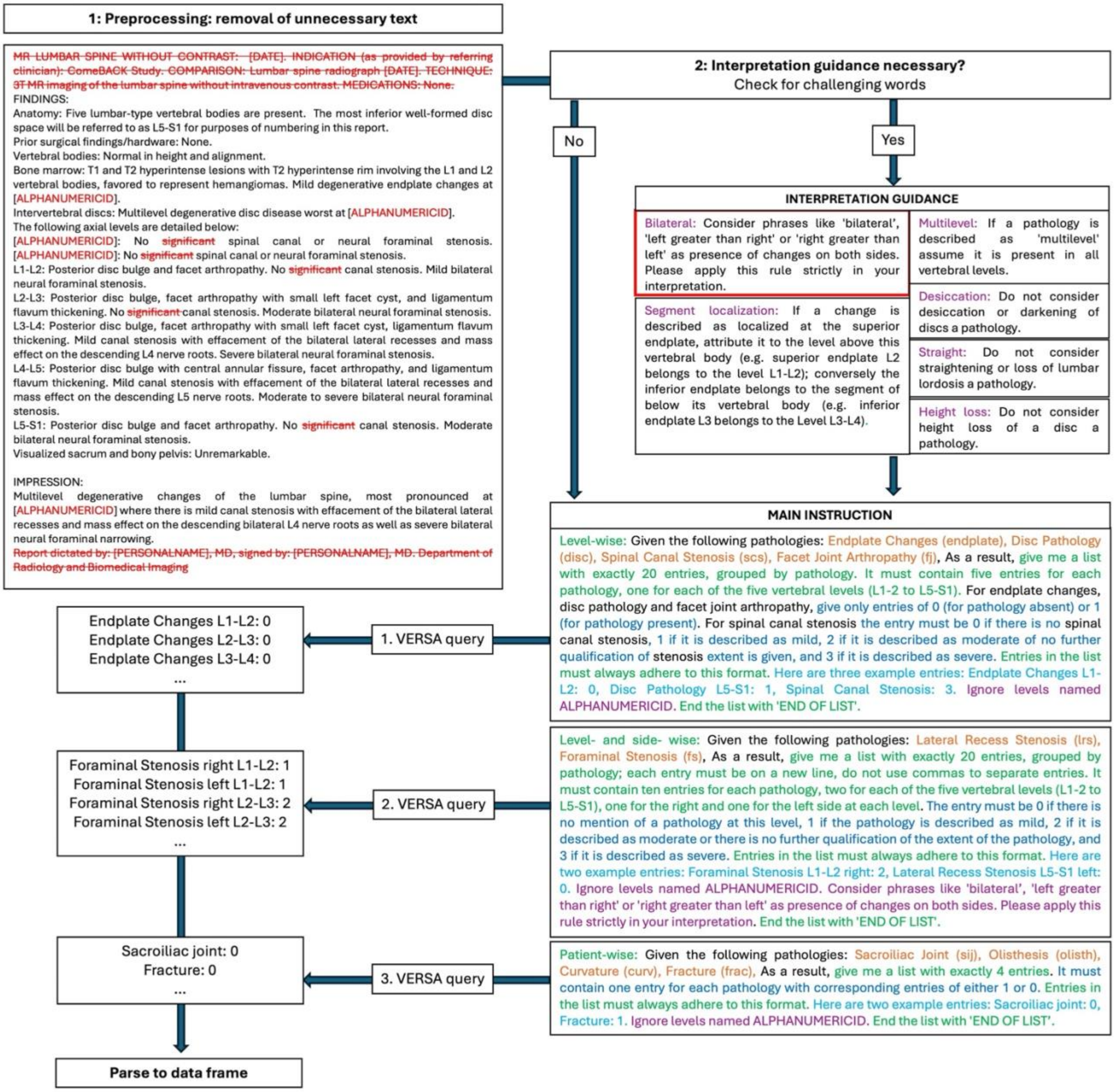

Supplemental Figure S1. Prompt assembly and pipeline schematic. *Preprocessing: Crossed out text is deleted (red). A simple search logic determines whether challenging wordings, identified in initial prompt development are present (purple) – in this example, the interpretation guidance that was used in the later prompt is framed in red. Main instruction: three different prompts are generated for each report, one for level-wise, one for level- and side-wise and one for patient-wise (green) pathologies (orange) with formatting instruction (dark blue) and few-shot examples (light blue). These are fed sequentially to VERSA, and their respective outputs are then parsed into a data frame.*
