# Supplementary material for: Information Extraction from Lumbar Spine MRI Radiology Reports Using GPT4: Accuracy and Benchmarking Against Research-Grade Comprehensive Scoring
Source: Diagnostics (Basel). 2025 Apr 4;15(7):930. doi: 10.3390/diagnostics15070930 (PMC11989208; doi:10.3390/diagnostics15070930)
Supplement: Supplementary file 1 [file diagnostics-15-00930-s001.zip › diagnostics-3560773-supplementary/Supplementary_Figure2.pdf]

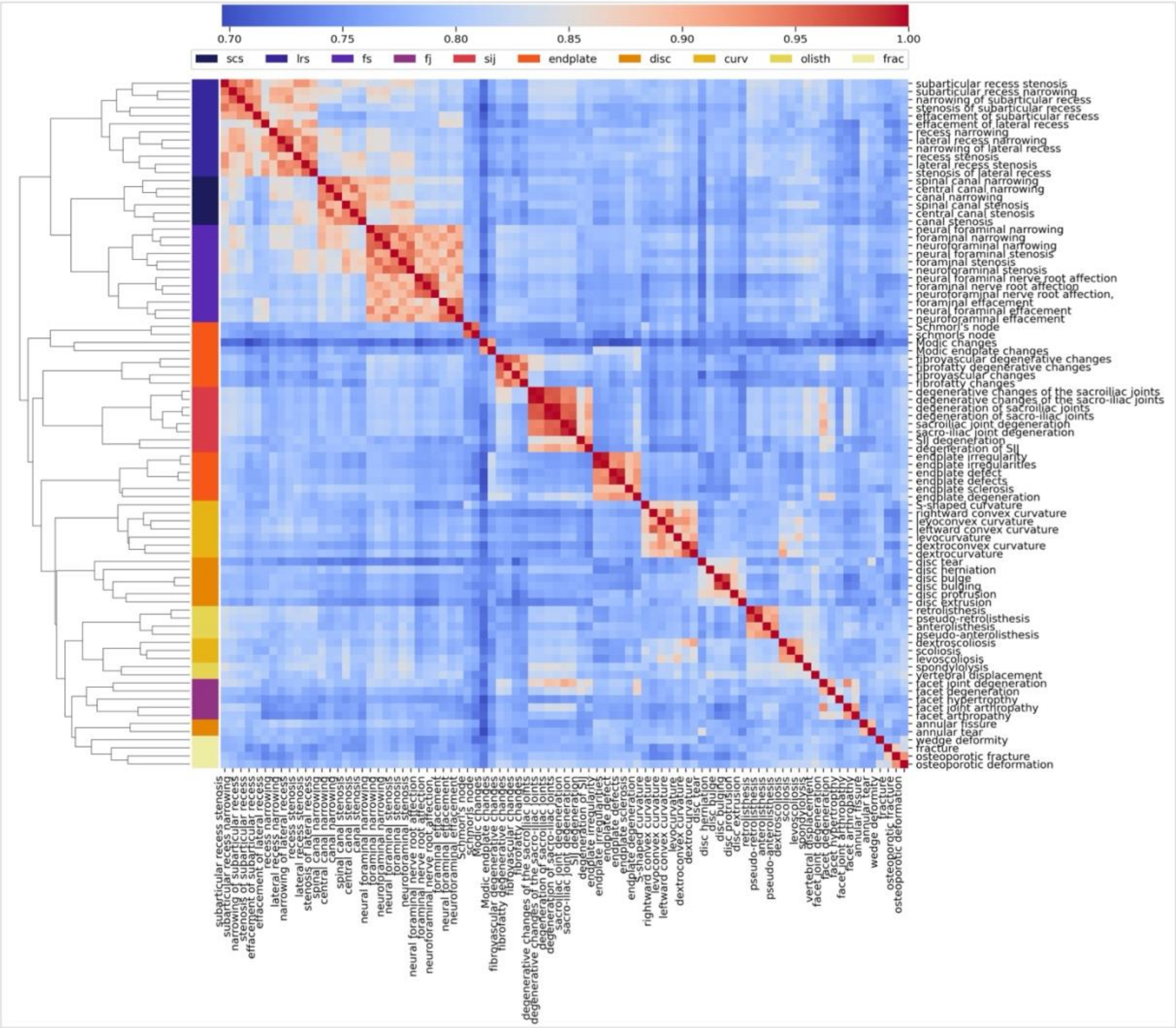

***Supplemental Figure S2. Heatmap of Cosine** Similarity between Embeddings of Pathology Terms. Scs=spinal canal stenosis. Lrs=lateral recess stenosis. Fs=foraminal stenosis. Sij=sacroiliac joint. Curv=pathology of the curvature of the spine. Olisth=olisthesis. Frac=fracture. Cosine similarities (red-blue) between each pair of each pathology term embeddings are shown in the heatmap with values of 1 indicating perfect similarity. Unsupervised agglomerative clustering of cosine similarities allowed for a data driven approach to understand model comprehension of term similarity. The color bar along the y-axis shows expert assigned pathology categories in comparison with data-derived categories. Distinct color bands (yellow-purple), such as for each type of stenosis, suggest the model can reconcile semantic variability when addressing the challenge of diverse medical terminology, thereby lending insight for prompt development*
